# Relative Sensitivity of Conventional and Real-Time PCR Assays for Detection of SFG *Rickettsia* in Blood and Tissue Samples from Laboratory Animals

**DOI:** 10.1371/journal.pone.0116658

**Published:** 2015-01-21

**Authors:** Galina E. Zemtsova, Merrill Montgomery, Michael L. Levin

**Affiliations:** Rickettsial Zoonoses Branch, Centers for Disease Control and Prevention, Atlanta, Georgia, United States of America; Institut National de la Recherche Agronomique, FRANCE

## Abstract

Studies on the natural transmission cycles of zoonotic pathogens and the reservoir competence of vertebrate hosts require methods for reliable diagnosis of infection in wild and laboratory animals. Several PCR-based applications have been developed for detection of infections caused by Spotted Fever group *Rickettsia *spp. in a variety of animal tissues. These assays are being widely used by researchers, but they differ in their sensitivity and reliability. We compared the sensitivity of five previously published conventional PCR assays and one SYBR green-based real-time PCR assay for the detection of rickettsial DNA in blood and tissue samples from *Rickettsia*- infected laboratory animals (n = 87). The real-time PCR, which detected rickettsial DNA in 37.9% of samples, was the most sensitive. The next best were the semi-nested *omp*A assay and *rpo*B conventional PCR, which detected as positive 18.4% and 14.9% samples respectively. Conventional assays targeting *omp*B, *glt*A and *hrt*A genes have been the least sensitive. Therefore, we recommend the SYBR green-based real-time PCR as a tool for the detection of rickettsial DNA in animal samples due to its higher sensitivity when compared to more traditional assays.

## Introduction

Studies on the natural transmission cycles of zoonotic pathogens and the reservoir competence of vertebrate hosts require methods for reliable diagnosis of infection in wild and laboratory animals. A variety of PCR-based assays are now widely used for the detection of rickettsial DNA in animal tissues and blood samples [1, 2, 3]. However, molecular assays are not infallible as the quality of the extracted DNA and, consequently, the PCR results are affected by conditions of sample acquisition, storage, and transportation, as well as methods used for DNA preparation. The presence of various PCR inhibitors, such as heme in whole blood samples, also affects performance of PCR assays [4, 5]. The choice of gene target for amplification, fitness and robustness of primers, and the degree of assay optimization also significantly affect the sensitivity and reliability of molecular assays. Researchers use different molecular methods for detection of SFG pathogens, but the obtained results are often incongruent and difficult to compare without knowing the relative sensitivity of test methods used in different studies.

Progress in developing real-time PCR assays opens new perspectives for improving diagnostic performance of molecular methods, particularly in regard to their sensitivity. The aim of the current study is to compare relative sensitivity of five of the previously published and commonly used conventional PCR assays and one SYBR green-based real-time PCR in detection of rickettsial DNA in blood and tissue samples from *Rickettsia*- infected laboratory animals.

## Methods

Blood and tissue samples were obtained from guinea pigs experimentally infected with *Rickettsia rickettsii* according to protocol 2555 approved by the Centers for Disease Control Institutional Animal Care and Use Committee (IACUC). Samples were collected during the course of clinical illness in the experimentally infected animals–between days 3 and 12 postinfection. A total of 87 samples (55 blood and 32 skin biopsy samples) were tested in parallel by each of the six assays described below. DNA was extracted from each sample using the Qiagen DNeasy Blood and the Tissue kit for skin samples and FlexiGene extraction kit (Qiagen Inc., Valencia, CA) for blood samples according to manufacturer’s protocols and eluted in 100ul (final volume) of elution buffers.

Overall, five conventional assays and one real-time PCR were performed on each sample (Table 1). Single step reactions amplifying *glt*A [6], *omp*B [7] and *rpo*B [8] gene targets, a semi-nested PCR targeting *omp*A gene [9,10], and a nested PCR detecting *hrt*A gene encoding 17kDa antigen [11, 12] were executed following the published protocols. The SYBR green-based real-time assay [13] targeting the same fragment of the *omp*A gene as the semi-nested assay was performed with a minor modification–the number of amplification cycles was limited to 40 instead of 50. Assays were completed on ABI 7500 thermocycler (Applied BioSystems, Foster City, CA) using Taq PCR Master Mix Kit (Qiagen Inc., Valencia, CA) and iCycler real-time detection system (Bio-Rad, Hercules, CA) using QuantiTect SYBR Green PCR Kits (Qiagen Inc., Valencia, CA) for conventional and real-time assays respectively. In the real-time assay all samples were run in duplicates; negative (distilled water) and positive (*Rickettsia massiliae* plasmid) controls were included into each run. The temperature dissociation curve was analyzed for each amplicon and only amplicons with the appropriate melting temperature (76.5±0.5°C) were considered as true positive.

**Table 1:** List of assays and primer pairs used for comparison analysis.

Assay	Gene target	Primers	№ of cycles	Reference
SYBR green-based qPCR	*omp*A	RR190–547 f	40	13
		RR190–701 r		
Nested PCR	*hrt*A	R 17–122 f	40	11
		R 17–500 r		
		TZ 15 f	30	12
		TZ16 r		
Semi-nested PCR	*omp*A	RR190–70 f	40	9
		RR190–701 r		
		RR190–70 f	30	10
		RR190–602 r		
PCR	*glt*A	CS-78 f	40	6
		CS-323 r		
PCR	*omp*B	120–2788 f	40	7
		120–3599 r		
PCR	*rpo*B	RPOB-FAV	40	8
		RPOB-RAV		

The data was analyzed using a Z test considering a P value ≤ 0.01 as statistically significant.

## Results

In total, 14 (25.5%) of the 55 blood samples were identified as positive by one or more assays. The SYBR green-based assay detected the presence of rickettsial DNA in 13 samples (23.6%) (Table 2). The semi-nested assay and a single step PCR targeting *omp*A and *omp*B genes respectively each detected rickettsial DNA in 2 of 55 (3.6%) samples. Conventional assays targeting *rpo*B and *glt*A genes and the nested assay targeting *hrt*A each detected rickettsial DNA in one of 55 (1.8%).

**Table 2:** Relative sensitivity of 5 conventional PCRs and SYBR green real-time PCR.

Sample type	Number of samples and percentage detected positive by each assay					
	qPCR	Semi-nested PCR	PCR	PCR	PCR	Nested PCR
	*omp*A	*omp*A	*rpo*B	*omp*B	*glt*A	*hrt*A
Blood	13/55	2/55	1/55	2/55	1/55	1/55
	(23.6%)	(3.6%)	(1.8%)	(3.6%)	(1.8%)	(1.8%)
Skin biopsy	21/32	14/32	12/32	5/32	4/32	3/32
	(65.6%)	(43.8%)	(37.5%)	(15.6%)	(12.5%)	(9.4%)
Total	34/87	16/87	13/87	7/87	5/87	4/87
	(39.1%)	(18.4%)	(14.9%)	(8%)	(5.7%)	(4.6%)

Out of the 14 positive blood samples, the presence of rickettsial DNA was detected in ten samples by a single assay only: in nine by the SYBR green-based assay and in one by the nested assay targeting *hrt*A only (Table 3). A total of four samples were positive in more than one assay. Three blood samples were concomitantly tested as positive by two assays–by real-time PCR and either the semi-nested PCR targeting *omp*A gene (2 samples) or the conventional PCR targeting *omp*B (1 sample). Only one blood sample was tested positive by four different assays, but was negative in either PCR targeting *rpo*B or the nested *hrt*A PCR (Table 3). Real-time PCR was the most sensitive assay for detecting SFGR in blood samples in comparison to each of the five conventional assays (p<0.01). There was no significant difference among all conventional PCRs with regard to detection of rickettsial DNA in blood samples.

**Table 3:** Incongruence of PCR results performed by 5 conventional PCRs and real-time PCR on positive animal blood samples.

Sample №	SYBR	Semi-nested	Single-step	Single-step	Single-step	Nested
	*omp*A	*omp*A	*rpo*B	*omp*B	*glt*A	*hrt*A
1	+	-	-	-	-	-
2	+	-	-	-	-	-
3	+	-	-	-	-	-
4	+	+	-	-	-	-
5	+	+	-	-	-	-
6	+	-	+	+	+	-
7	+	-	-	-	-	-
8	-	-	-	-	-	+
9	+	-	-	-	-	-
10	+	-	-	+	-	-
11	+	-	-	-	-	-
12	+	-	-	-	-	-
13	+	-	-	-	-	-
14	+	-	-	-	-	-

Altogether, the six compared assays detected rickettsial DNA in 22 of 32 (68.8%) skin biopsy samples. The SYBR green real-time PCR identified 21 (65.6%) of those samples, the semi-nested *omp*A targeting PCR identified rickettsial DNA in 14 (43.8%), and PCR targeting *rpo*B recognized 12 (37.5%) of 32 samples as positive (Table 2). On the other hand, only 5 (15.6%), 4 (12.5%) and 3 (9.4%) out of 32 skin biopsy samples were tested positive by molecular assays targeting *omp*B, *glt*A and *hrt*A genes respectively (Table 2).

The detection consistency across assays was better for skin biopsies than for blood samples (Table 4). Only three out of the 22 positive skin samples were identified by the SYBR real-time PCR alone, and one out of 22 samples was detected as positive by the conventional assay targeting *rpo*B gene, but not by any other assay. Presence of rickettsial DNA was detected by multiple methods in 18 skin samples: 7/18, 6/18, 3/18 and 2/18 samples were detected positive by 2, 3, 4 and 5 assays respectively (Table 4). There was no statistically significant difference in the number of positive tests between the SYBR real-time PCR and assays targeting *omp*A or *rpo*B genes; however, qPCR assay was found significantly more sensitive in testing skin samples compared to conventional assays targeting *omp*B, *glt*A, or *hrt*A genes (p<0.01).

**Table 4:** Incongruence of PCR results performed by 5 conventional PCRs and real-time PCR on positive animal skin biopsy samples.

Sample №	SYBR	Semi-nested	Single-step	Single-step	Single-step	Nested
	*omp*A	*omp*A	*rpo*B	*omp*B	*glt*A	*hrt*A
1	+	-	-	-	-	-
2	+	-	-	+	-	-
3	+	+	+	-	-	-
4	+	-	-	-	-	-
5	+	+	+	-	+	-
6	+	+	+	-	-	-
7	+	-	+	-	-	-
8	+	+	-	-	+	+
9	+	+	+	+	-	-
10	+	+	-	+	+	+
11	+	-	-	-	-	-
12	+	+	+	+	-	-
13	+	+	+	-	-	-
14	+	+	+	-	-	-
15	-	-	+	-	-	-
16	+	+	+	-	-	-
17	+	+	-	-	-	-
18	+	+	+	+	+	-
19	+	-	-	-	-	+
20	+	-	+	-	-	-
21	+	+	-	-	-	-
22	+	+	-	-	-	-

Overall, the SYBR green-based qPCR assay detected presence of rickettsial DNA in 34 (39.1%) of 87 blood and tissue samples. The next best were the semi-nested *omp*A assay and the conventional *rpo*B PCR, which detected 16 (18.4%) and 13 (14.9%) out of 87 samples respectively. Conventional PCR assays targeting *omp*B, *glt*A and *hrt*A genes had been the least sensitive detecting rickettsial DNA in only 7 (8%), 5 (5.7%) and 4 (4.6%) out of 87 samples respectively. The sensitivity difference between qPCR assay and all other assays analyzed in this study was found to be statistically significant (p<0.01).

## Discussion

In the current study, we compared the relative sensitivity of five conventional and one real-time SYBR-green based qPCR assays intended for detection of SFGR in animal-derived skin biopsies and blood samples. The evaluated assays are genus-specific and do not differentiate among different *Rickettsia* species. The sensitivity of all assays, which directly affects results of animal model studies, was compared using *R*. *rickettsii* as the target. Our results are similar to those reported by Barrett and colleagues [3] who compared the efficiency of three conventional assays for detection of rickettsial DNA in whole blood samples obtained from dogs. In their study, assays targeting *glt*A, *hrt*A and *omp*A genes detected rickettsial DNA in 0 percent, 2.4% and 11.5% respectively. Curiously, both the present study and the one by Barrett and colleagues [3] found the single-stage assay detecting *glt*A gene among the least sensitive, whereas Santibanez and colleagues [14] appraised a *glt*A nested assay with the same primers for the primary reaction as the most sensitive. The latter group had not considered a real-time PCR because of its reputed high cost and limited availability [14]. However, development and utilization of molecular applications are progressing extremely quickly; such methods are becoming cheaper and more affordable for laboratories. Because of its higher sensitivity and rapidity, the real-time PCR should be considered an alternative to conventional molecular assays routinely used in animal studies. Thus, we intentionally compared conventional PCR methods to the real-time PCR and demonstrated statistically higher sensitivity of the latter. Notably, there was a somewhat higher degree of discrepancy between the real-time and conventional assays in testing blood samples versus skin samples. This may be due to either higher concentrations of target DNA in skin making its detection easier, or better ability of the assays to overcome inhibiting effects of heme remaining in DNA extracted from whole blood, or both.

An approach using a series of 2–3 independent reactions may increase detection of the target DNA but makes testing much more expensive, time- and labor-consuming. This approach also creates additional risk for laboratory errors and a potential source of contamination. Detection of rickettsial DNA by conventional methods in two samples, which tested negative in the qPCR assay, demonstrates that even the latter does not possess the absolute sensitivity. It, however, does not alter the fact that the real-time method reliably detected presence of SFG rickettsial DNA in greater proportion of animal blood and skin samples than any of the five compared conventional methods.

Considering that all blood and skin samples used for this evaluation were taken from laboratory animals known to be infected with *R*. *rickettsii*, it was expected that a large percentage of samples would contain some rickettsial DNA, especially those collected at the peak of infection. However, the distribution of the pathogen, dynamics of infection and the bacterial load in different types of samples have not been validated yet. Therefore, a low quantity of target DNA in the samples might be the major obstacle for pathogen detection by different methods. Although caution is recommended in reporting sensitivity of real-time in comparison to conventional PCR in each particular case [15], the presented results demonstrate better performance of the real time assay for detection of rickettsial agents. The real-time PCR considered here is a SYBR green-based assay for which all required validation procedures have been performed, limit of detection was determined and the additional amplicon melting temperature analysis step was incorporated to account for the specificity-related concerns often associated with SYBR green-based assays [13]. However, we limited the number of amplification cycles in this assay to 40 instead of the originally recommended 50 in order to minimize possible non-specific amplification as well as to further equalize all assays being compared. Thus, the greater proportion of positive detections by the real-time assay can be attributed to higher sensitivity, not due to an increased number of amplification cycles. The sensitivity of the described assay (5 copies of DNA per 25 uL reaction) is comparable with that of another SYBR green-based assay detecting *omp*A gene target developed by Kidd and colleagues [16]. Our results are also in accordance with those validated by another group that developed a novel TaqMan real-time PCR for detection of *Rickettsia* spp. in clinical specimens and assessed its performance against traditionally used assays [17]. Considering all aforementioned factors, we recommend employing a real-time PCR as a tool for detection of rickettsial DNA in samples obtained from laboratory or wild animals, especially those that contain a low number of DNA copies, due to its higher sensitivity when compared to several of the traditionally used assays. Further development of rapid, sensitive, and affordable molecular applications that can be used as standard diagnostic methods is worthy of investment of resources.
